# Foliar mycobiome remains unaltered under urban air-pollution but differentially express stress-related genes

**DOI:** 10.1007/s00248-024-02387-y

**Published:** 2024-05-17

**Authors:** Valeria Stephany Flores-Almaraz, Camille Truong, Diana Hernández-Oaxaca, Verónica Reyes-Galindo, Alicia Mastretta-Yanes, Juan Pablo Jaramillo-Correa, Rodolfo Salas-Lizana

**Affiliations:** 1 Posgrado en Ciencias Biológicas, Unidad de Posgrado, Edificio A, 1° Piso, Circuito de Posgrados, Ciudad Universitaria, Coyoacán, C.P. 04510 Distrito Federal, México; 2 Instituto de Biología, Universidad Nacional Autónoma de México, Av. Universidad 3000, 04510 Coyoacán, Ciudad de México Mexico; 3 Royal Botanic Gardens Victoria, Birdwood Ave, Melbourne, VIC 3004 Australia; 4 Centro de Ciencias Genómicas, Universidad Nacional Autónoma de México, Av. Universidad S/N, 62210 Cuernavaca, Morelos México; 5 Depto. de Ecología Evolutiva, Instituto de Ecología, Universidad Nacional Autónoma de México, Av. Universidad 3000, 04510 Coyoacán, Ciudad de México Mexico; 6 Consejo Nacional de Humanidades Ciencias y Tecnología (CONAHCYT), Avenida Insurgentes Sur 1582, Crédito Constructor, Benito Juárez, Ciudad de México, 03940 México; 7 Departamento de Ecología de la Biodiversidad, Instituto de Ecología, Universidad Nacional Autónoma de México, Av. Universidad 3000, 04510 Coyoacán, Ciudad de México Mexico; 8 Laboratorios de Micología. Depto. de Biología Comparada, Facultad de Ciencias., Universidad Nacional Autónoma de México, Circuito Exterior s/n, Ciudad Universitaria, Coyoacán, 04510 Ciudad de México, México

**Keywords:** Metatranscriptomics, Metabarcoding, Oxidative stress, Tropospheric ozone, Foliar endophytes, *Abies religiosa*

## Abstract

Air pollution caused by tropospheric ozone contributes to the decline of forest ecosystems; for instance, sacred fir, *Abies religiosa* (Kunth) Schltdl. & Cham. forests in the peri-urban region of Mexico City. Individual trees within these forests exhibit variation in their response to ozone exposure, including the severity of visible symptoms in needles. Using RNA-Seq metatranscriptomic data and ITS2 metabarcoding, we investigated whether symptom variation correlates with the taxonomic and functional composition of fungal mycobiomes from needles collected in this highly polluted area in the surroundings of Mexico City. Our findings indicate that ozone-related symptoms do not significantly correlate with changes in the taxonomic composition of fungal mycobiomes. However, genes coding for 30 putative proteins were differentially expressed in the mycobiome of asymptomatic needles, including eight genes previously associated with resistance to oxidative stress. These results suggest that fungal communities likely play a role in mitigating the oxidative burst caused by tropospheric ozone in sacred fir. Our study illustrates the feasibility of using RNA-Seq data, accessible from global sequence repositories, for the characterization of fungal communities associated with plant tissues, including their gene expression.

**Supplementary Information:**

The online version contains supplementary material available at 10.1007/s00248-024-02387-y.

## Introduction

Tropospheric ozone (O_3_) is a serious threat to human health, biodiversity, and ecosystem function [1]. However, although the effects of O_3_ have been widely studied on plants [2], the impacts of O_3_ on fungal mycobiomes and interactions with stressed host plants are poorly understood. The fungal foliar mycobiome comprises fungi living on the surface of leaves (epiphytes) and those inhabiting inner tissues (endophytes). However, this division is not always evident, as some endophytes may live as epiphytes for some part of their life cycle, and in the practice it is common that some epiphytes are still present after leaves surface sterilization [3]. Endophyte fungi have received more attention, and it is likely that the foliar mycobiome is mostly constituted by foliar endophytes. Fungal endophytes are plant-inhabiting fungi that do not produce symptoms of colonization in their hosts for most of their life cycle [4]; they may exhibit a broad spectrum of life-history strategies, from parasitic to mutualistic [5]. When acting as mutualists, these fungi can enhance their host’s fitness, when they face biotic and abiotic stresses, including oxidative stress [6].

The taxonomic and functional composition of fungal communities in leaves can be affected by several biotic and abiotic stressors. For example, fungal pathogens and bacteria can cause the regreening of senescent leaf tissues in *Acer*, while reducing the overall fungal richness and altering leaf fungal communities [7]. In contrast, fungal endophyte communities are not correlated with pathogenic symptoms in *Vanilla* or *Eucalyptus* plants, respectively affected by pathogenic fungi and a wasp [8, 9]. However, in the case of *Eucalyptus*, differentially expressed genes associated with secondary metabolism and fungal biomass were observed between resistant and susceptible plants [8]. Regarding O_3_ stress, Liu et al*.* [10] found a general decrease in overall fungal richness and an increase in pathogenic fungi abundance in *Euonymus japonicus* plants experimentally exposed to high levels of O_3_. Other studies have suggested that fungal endophytes may minimize the extent of O_3_ damage in their plant hosts by secreting antioxidant agents or inducing the activation of plant antioxidants [11]. However, no study so far has investigated the effect of O_3_ on the gene expression of foliar mycobiomes.

**Fig. 1:**
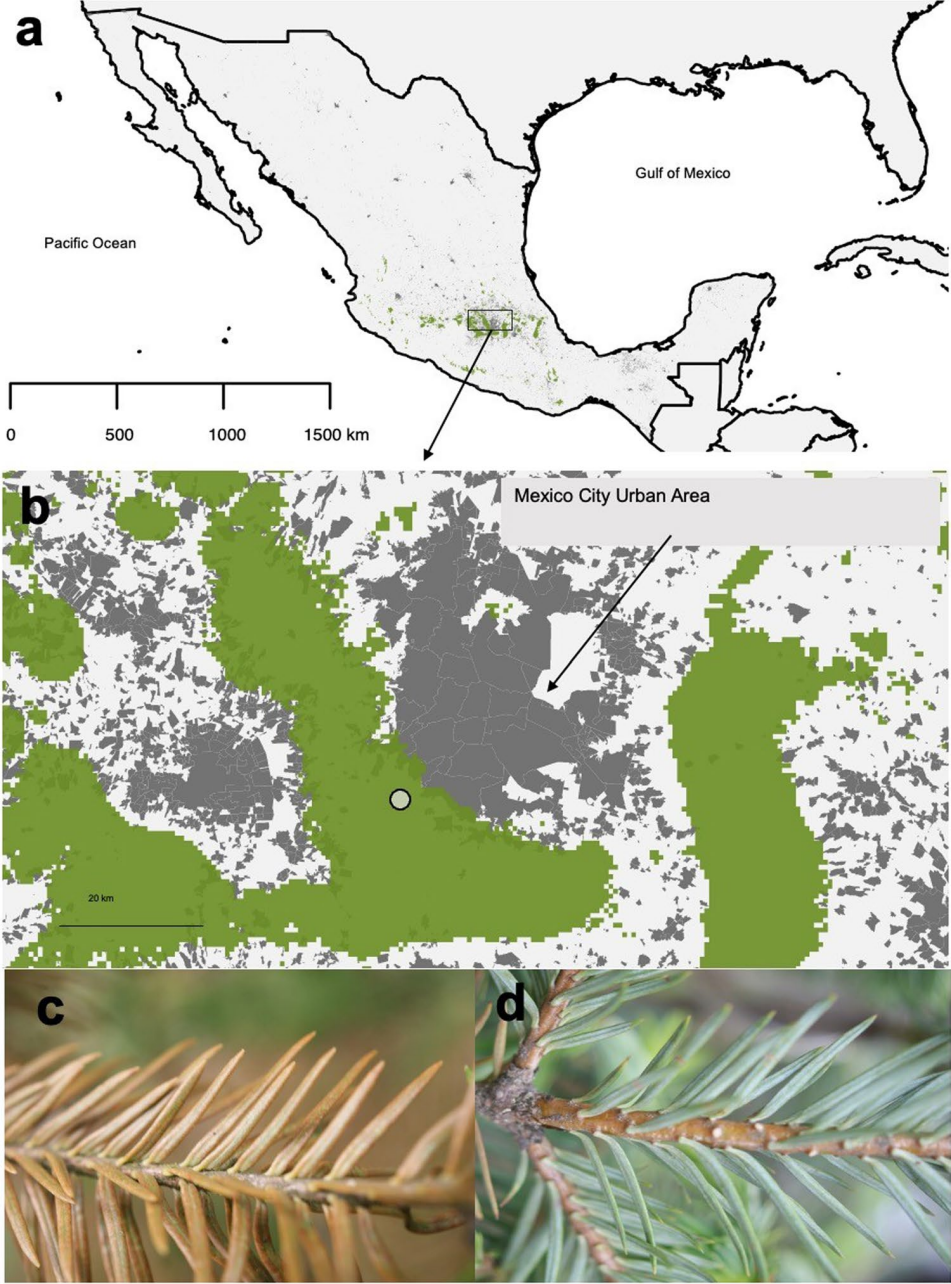
**a** Distribution of *Abies religiosa* in Mexico; **b** Approximate distribution of the species (green areas; taken from [18]) and neighboring urban and rural zones (gray areas; according to INEGI [19]) in central Mexico. The circle in the forest area depicts the approximate location of the study site; **c** symptomatic needles (note the reddish tones); and **d**, asymptomatic needles

Tropospheric ozone is a major concern in heavily populated cities, like Mexico City. The geographic location and topography of Mexico City, which is situated above 2,200 m asl and encircled by mountains in three directions, facilitates the concentration of high amounts of O_3_ for extended periods of time [12], and exacerbates its detrimental effects on urban and peripheral forests [13, 14]. Air pollutants in Mexico City drain across the surrounding mountains, particularly towards the southwest, where sacred fir forests (*Abies religiosa* (Kunth) Schltdl. & Cham.) dominate the native vegetation (Fig. 1a-b). In regions such as the Desierto de los Leones National Park, situated ca. 30 km southwest of Mexico City, O_3_ concentrations can be as high as 161 ppb between March and June (the dry-warm season) [15]. These values exceed by five times the recommended level for safeguarding forest trees [16]. In firs from these forests, observed symptoms related to O_3_ stress include leaf chlorosis, needle casting, branch loss, and even death [17].

Sacred fir is a conifer tree native to central and southern Mexico and western Guatemala [20], where it forms monodominant forests (Fig. 1a-b). These forests represent one of the few remaining forested areas in the vicinity of Mexico City, where they provide ecosystem services, such as erosion control, carbon sequestration and water retention [21]. Most of the remaining forests are conserved within Desierto de los Leones National Park, Santa Rosa Xochiac communal land and the territories of other local communities’ [22]. In sacred firs, external visible signs of O_3_ damage initially appear as faint whitish spots on the upper surface of needles, gradually coalescing and developing into larger lesions with brownish-red coloration [23] (Fig. 1c-b). Despite the elevated O_3_ levels at Desierto de los Leones National Park, external needle symptoms can vary among individuals, with some individuals displaying symptoms in several, but not all branches, while other trees remain completely asymptomatic [23]. The presence of external symptoms was associated with distinct terpenoid profiles and gene expression patterns [14]. Specifically, asymptomatic trees produce some sesquiterpenes related to oxidative stress response, like β-pinene [24]. Differences between asymptomatic and symptomatic trees have been also found at the gene-expression level, with transcripts related to stomatal opening and response to stress being up-regulated in asymptomatic trees [24].

Similar responses to O_3_ stress have been extensively documented in other plants in both natural settings and controlled experiments [25–27]. However, a comprehensive understanding of the physiological effects of O_3_ in plant-associated fungal communities, as well as its impact on the interactions between plants and their associated mycobiota, remain poorly studied. Several studies on the effects of O_3_ on fungi have focused on its use as antifungal agent for seed sterilization and for controlling food contamination caused by *Aspergillus*, *Penicillium*, and *Rhizopus* [28, 29]. The antifungal effect of O_3_ is attributed to its ability to increase fungal cell membrane permeability, which induces cell wall damage, and thereby alter lipid, carbohydrate, and protein metabolisms [29]. These alterations ultimately lead to the excessive accumulation of reactive oxygen species (ROS) and death [28].

RNA-seq data is an increasingly important tool for plant studies on development, stress response, and biotic interactions, among others [30, 31]. RNA extracted from plant tissues most likely contains RNA from the associated microbiome (fungi and bacteria), allowing the study of the gene expression of plant microbiomes [32]. For example, Chialva et al. [33] mined previously generated RNA-Seq data from tomato plants to infer the taxonomy and function of the root microbiota. In this study, we characterized the fungal communities and fungal gene expression from O_3_-related symptomatic and asymptomatic *A. religiosa* needles using ITS2 metabarcoding and reanalyzing previously generated plant RNA-Seq data [24]. We hypothesized that: 1) the presence of visible O_3_-associated symptoms is correlated with changes in the taxonomic composition of fungal communities within *A. religiosa* needles, leading to a reduction in species richness and their relative abundance; and 2) the mycobiome present in asymptomatic needles differentially express genes associated with antioxidant mechanisms. Finally, we evaluated whether RNA-Seq data targeting host plants could provide significant taxonomic and functional information about the fungal communities of these plants.

## Methods

### Sampling and data generation

Samples used in this study were previously collected and processed by Reyes-Galindo [24] in a natural *Abies religiosa* population. Briefly, we collected samples at the Santa Rosa Xochiac community (19.285 N, 99.301 W), in the buffer zone of the Desierto de los Leones National Park, southwest of Mexico City, in May 2017. We selected this area due to the elevated mortality rate of *A. religiosa* individuals, which can be attributed to a combination of factors, but especially air pollution [34].

We collected needles from five 10–15 years old individuals with reddish needles (i.e., symptomatic) and five other individuals of the same age without visible symptoms (i.e., asymptomatic; Fig. 1 c, d); all trees were located within an area of approximately 1 ha. We collected symptomatic needles (Fig. 1c) from symptomatic trees and asymptomatic needles (Fig. 1d) from asymptomatic trees. In both cases we collected needles on branches from the previous two growth seasons (2015 and 2016), which were pooled, preserved in RNAlater (Sigma–Aldrich) and stored at -70 °C until processing. The needles were not surface sterilized.

For each sample, we extracted total RNA from 40–45 mg of plant tissue (4–5 needles) using the Spectrum RNA Plant Kit (SIGMA) and evaluated its integrity via 1% agarose gel electrophoresis. The quality and purity of the RNA were determined using a NanoDrop spectrophotometer based on the 260/280 and 260/230 ratios. Quantification of the RNA concentration was performed using the Qubit RNA assay (Invitrogen). Library construction was performed through mRNA enrichment by polyadenylated RNA capture (polyA-capture), retrotranscription, and subsequent sequencing using a HiSeq 4000 150 × 2 paired-end protocol (Illumina). The library preparation and sequencing procedures were carried out at the University of Berkeley, USA. Demultiplexing was performed by the sequencing service.

DNA was extracted from 80 mg of tissue ground with liquid nitrogen using a DNeasy Plant Mini Kit (QIAGEN), following the manufacturer's protocol. We amplified the ITS2 region of nuclear rDNA using the fungal-specific primers gITS7ngs and ITS4ngsUNI [35] with the Platinum PCR SuperMix High Fidelity Kit (Invitrogen). PCR conditions were as follows: initial denaturation at 94 °C for 1 min, followed by 35 cycles at 94 °C for 30 s, 52 °C for 30 s, 68 °C for 30 s, and a final extension step at 68 °C for 7 min. We constructed Amplicon libraries with Nextera adapters (Illumina), which were normalized at equimolar concentrations, purified with Agencourt AMPure XP beads (Beckman) and sequenced using a MiSeq 300 × 2 paired-end protocol (Illumina) at the University of Arizona Genetics Core, USA.

### ITS2 metabarcoding

We ‘denoised’ sequences and clustered them into operational taxonomic units (OTUs) at 97% similarity using the AMPtk 1.3.0 pipeline [36] following Bermúdez-Contreras et al. [37]. Best practice meta-barcoding protocols currently recommend OTU clustering of fungal ITS sequences over haplotype-based (Amplicon Sequence Variant, or ASV) approaches, due to the high variability of the non-coding ITS region, as well as evidence for intragenomic variability between ITS copies within an individual [38–40]. OTU taxonomy was assigned in AMPtk by aligning reference sequences with the UNITE database [41]. We used negative and positive controls according to Nguyen et al. [42]. We removed OTU occurrences that accounted for < 0.5% of sequence counts per sample to eliminate potential sequencing artifacts. To account for the variation in the number of ITS2 reads per sample, we transformed read counts into relative abundances by averaging the number of reads per sample, multiplying them by 1000 and transforming them to the next integer, which was used as counts [43].

### RNA-Seq metatranscriptomics

We used *Abies religiosa* RNA-Seq data generated from Reyes-Galindo [24]. We removed low quality reads and adapters using Trimmomatic v0.39 [44] and verified read quality using FastQC v0.11.8 [45] and MultiQC v1.0.dev0 [46]. We removed the host reads by mapping the reads to the *Abies balsamea* transcriptome (GenBank Accession: GGJG00000000, Bioproject: PRJNA437248) using the BWA-MEM software [47]. We then assembled both paired and unpaired reads that did not map to the reference transcriptome into contigs using SPAdes v3.13.0 [48], and estimated assembly statistics with QUAST v5.0.2 [49].

Additionally, to maximize the number of assembled genes for functional analyses, we produced five de novo assemblies using host-filtered RNA-Seq reads from all samples: one with rnaSPAdes v3.13.0 with default parameters, and four with Trinity v2.8.5 [50] using the parameters specified in Table S1. We evaluated assembly quality by measuring the number of genes and isoforms with RSEM v1.3.3 [51], and assembly length with metaquast v3.2 [52]; we then selected the assembly providing the highest number of assembled genes, isoforms, and total length.

To taxonomically assign RNA-Seq reads, we implemented two widely used algorithms in shotgun metagenomic studies for fungal taxonomic classification: Kraken2 v.2.1.2-Bracken [53, 54] and Kaiju v1.8.0 [55]. We used the RefSeq database, limiting the search parameters to complete fungal genomes/proteins, and performed both analyses using high-quality filtered reads and contigs separately. We evaluated the performance of both algorithms and data types by counting the number of identified taxa at each taxonomic rank and comparing them to the number of identified taxa using ITS2 metabarcoding. The database-algorithm combination with the smallest ratio of unique taxa was selected for further analyses.

### Taxonomic profiling

We characterized and visualized fungal communities in R v4.0.2 [56] using the phyloseq v.1.44.0 [57], ggvenn v0.1.9 [58], microbiome v1.13.12. [59], vegan v2.5–7 [60], eulerr v6.1.1 [61], ggplot2 v3.4.2 [62], DESeq2 v.40.1 [63], indicspecies v1.7.13 [64], Ampvis2 v2.8.3 [65], file2meco v.0.7.0 [66] and microeco v.1.4.0 [67] packages. Guilds were assigned to OTUs based on FungalTraits [68], using the “Primary lifestyle” classification. All analyses were performed separately for each dataset: ITS2 metabarcoding and RNA-seq metatranscriptomics data. To evaluate differences in community attributes between symptomatic and asymptomatic needles, we used the following response variables: 1) taxonomic community composition; 2) association of specific OTUs to conditions; 3) OTUs observed richness; 4) class relative abundance and 5) guild composition.

We assessed differences in community composition between symptomatic and asymptomatic needles using a PERMANOVA (permutational analysis of variances) with 999 permutations (alpha = 0.05) and Raup-Crick distances, based on OTU presence/absence, after eliminating OTUs that were only present in one sample. Community composition was visualized using non-metric multidimensional scaling (NMDS). To evaluate associations between the relative abundance of specific OTUs and the presence of symptoms, we calculated the indicator value index (IndVal) [69] and assess statistical differences with 999 permutations (alpha = 0.05). We tested for differences in community observed richness between needle condition fitting generalized linear models with a Poisson distribution and a log-link function. We evaluated the fit of our models by comparing them to null models using chi-square tests (alpha = 0.05). To test for differences in the relative abundance of fungal classes between asymptomatic and symptomatic needles, we calculated the log_2_FoldChange using the symptomatic condition as reference and conducted Wald tests with adjusted *p-values* for multiple comparisons using the Benjamini–Hochberg method (alpha = 0.05), as implemented in DESeq2. To compare guild composition between symptomatic and asymptomatic needles, we performed a PERMANOVA with 999 permutations (alpha = 0.05) based on a Bray–Curtis distance matrix.

### Functional profiling

We predicted open reading frames (ORFs) with Transdecoder v.5.5.0 [70], annotated them with eggNOG-mapper v2 [71] against the eggNOG 5.0 database [72], and retained only fungal ORFs that were assigned to at least one Cluster of Orthologous Groups (COG) [73]. We estimated transcription levels by mapping the host-filtered reads to the predicted ORFs using Salmon v1.8.0 [74] and imported them into R v4.0.2 using the tximport package [75]. After removing ORFs with less than 10 counts, we evaluated statistically significant differences in log_2_FoldChange of ORFs using the symptomatic needles as reference, by performing Wald tests with adjusted *p-value* for multiple comparisons using the Benjamini–Hochberg method, as implemented in DESeq2. We considered a significant difference in transcription when log_2_FoldChange was smaller than -1 or greater than one and the *adjusted p-value* < 0.10. To designate ORFs to specific metabolic pathways or modules, we first assigned KEGG Orthology (KO) [76] numbers to each ORF using the KASS platform with the BLAST algorithm [77], and then we searched for KO numbers in the KEGG mapper [78], which renders all the associated metabolic pathways and modules for each query. Functional composition of COGs was visualized using non-metric multidimensional scaling (NMDS) based on Bray–Curtis distances among samples. To test for differences in dispersion between conditions, we used a betadispersion model and performed permutational analysis of multivariate dispersions (PERMDISP) with 999 permutations (alpha = 0.05). We assessed differences in the community composition of COGs between symptomatic and asymptomatic needles using a PERMANOVA (permutational analysis of variances) with 999 permutations (alpha = 0.05).

Custom scripts for all performed analyses are available at https://github.com/valeriafloral/Abies_foliar_mycobiome

## Results

### Taxonomic profiling

ITS2 metabarcoding produced 59,698–156,065 reads per sample, which clustered into 259 OTUs. In turn, between 89.2% and 95.8% of the quality-filtered RNA-Seq reads matched to *A. religiosa* which, after removal, yielded 12,500,000–21,200,000 nonhost reads per sample; these were assembled into 4,988–20,572 contigs per sample. The Kraken2-Bracken algorithm yielded 24,108–105,092 nonhost reads and 1,485–5,545 contigs that were taxonomically classified into 86 OTUs. The Kaiju algorithm allowed the identification of more taxa, for a total of 482 OTUs inferred from contigs and 484 from reads (Fig. S1). This algorithm also produced a higher ratio of uniquely identified taxa than Kraken2-Bracken (Fig. S2). We thus selected the Kraken2-Bracken dataset (based on reads) for all subsequent analyses.

### Diversity analyses

**Fig. 2:**
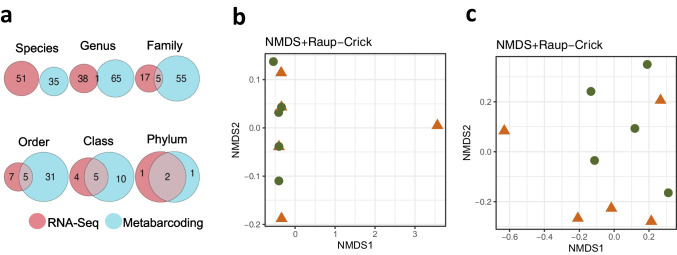
Taxonomic characterization of the mycobiome of needles from symptomatic (orange triangles) and asymptomatic (green dots) *Abies religiosa* trees (condition) using ITS2 metabarcoding and RNA-seq metatranscriptomics (dataset). **a** Venn diagrams showing the number of shared classified taxa between datasets at each taxonomic level. Nonmetric multidimensional scaling (NMDS) based on Raup–Crick distances among samples for **b** RNA-Seq metatranscriptomics (stress = 0.000092, PERMANOVA R^2^ = 0.003, F_(1, 8)_ = 0.027, *p-value* = 0.768); and **c** ITS2 metabarcoding (stress = 0.14, PERMANOVA R^2^ = 0.12, F_(1, 8)_ = 1.16, *p-value* = 0.53) datasets

A greater number of OTUs was detected when using ITS2 metabarcoding (n = 259) than when using RNA-Seq metatranscriptomics (n = 86). Both methods further retrieved different numbers of taxa at each taxonomic level; indeed, the two datasets barely overlapped, sharing no species, only one genus, five families, five orders, five classes and two phyla (Fig. 2a). We did not find significant differences in the taxonomic composition of fungal communities from symptomatic and asymptomatic needles in any dataset (*p-value* > 0.05). This can be seen in the results of the NMDS analysis for RNA-seq dataset (stress = 0.000092, Fig. 2b) and metabarcoding dataset (stress = 0.14, Fig. 2c), where no clear clusters were formed. Using the ITS2 metabarcoding dataset, we observed two OTUs significantly associated with asymptomatic needles, identified as Lecanorales sp. (IndVal = 0.89, *p-value* = 0.048) and *Phaeomoniella* sp. (IndVal = 0.89, *p-value* = 0.047), both within Ascomycota. No significant OTUs associated with the presence of symptoms in needles were found when using the RNA-Seq metatranscriptomic dataset (p > 0.05).

**Fig. 3:**
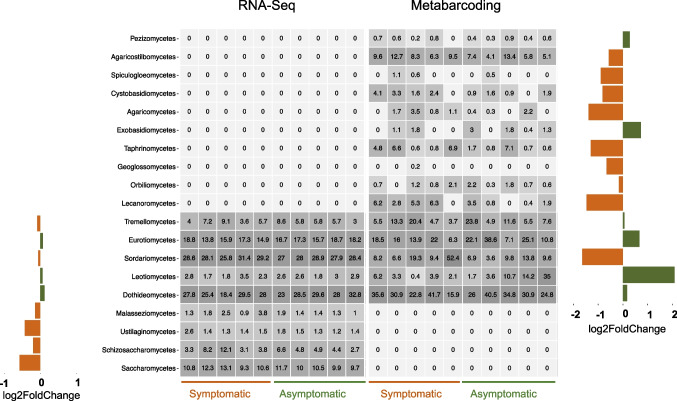
Taxonomic abundance of fungal classes within the mycobiome of needles from symptomatic and asymptomatic *Abies religiosa* trees determined from ITS2 metabarcoding and RNA-seq metatranscriptomic data. Heatmap depicts the relative abundance (expressed in percentage) of each identified fungal class (rows) per sample (columns). Bar plots on the sides show differences in relative abundance changes (represented as log_2_FoldChange) between needles from symptomatic (orange bars) and asymptomatic trees (green bars)

Similarly, there were no observed differences in species richness between needles from asymptomatic and symptomatic needles in either the RNA-Seq metatranscriptomics (χ^2^_1_ = 0.0, *p-value* = 1) or the ITS2 metabarcoding datasets (χ^2^_1_ = 1.01, *p-value* = 0.3). Although each dataset retrieved a different number of fungal classes (15 for ITS2 metabarcoding and nine for RNA-Seq metatranscriptomics), they shared the five fungal classes with the highest relative abundance, including Dothideomycetes, Eurotiomycetes, and Sordariomycetes (Fig. 3). None of the classes presented a statistically significant difference in their relative abundance between asymptomatic and symptomatic needles in any dataset (*adjusted p-value* > 0.05). However, class Leotiomycetes was marginally more abundant in asymptomatic needles (*p-value* = 0.06) when using ITS2-metabarcoding data.

For guild assignment, 87.6% of all OTUs were assigned using RNA-Seq metatranscriptomic data, from which the most abundant guild was plant pathogen (22 OTUs), while wood saprotroph was the least abundant (one OTU). For the ITS2 metabarcoding data, we assigned 32.8% of all OTUs. Plant pathogen was again the most abundant guild (34 OTUs), while epiphyte, ectomycorrhizal, and pollen saprotroph were the least abundant (one OTU for each guild) (Fig. S3). We further confirmed the identity of these OTUs with manual BLAST searches in NCBI (data not shown). We did not find significant differences in guild composition between asymptomatic and symptomatic needles (p > 0.05) for any of the datasets.

### Functional profiling

**Fig. 4:**
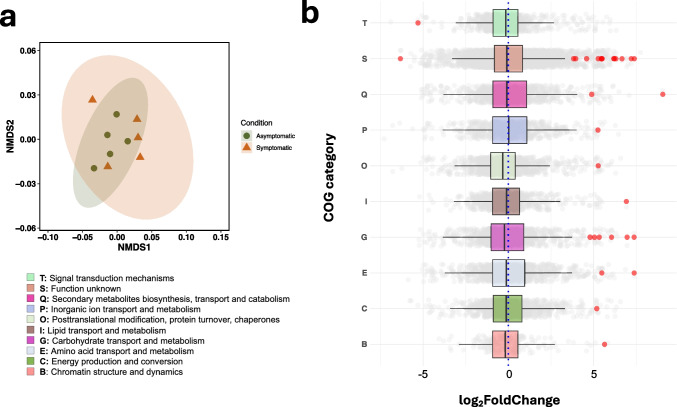
Mycobiome functional diversity in symptomatic (orange triangles) and asymptomatic (green circles) *Abies religiosa* needles. **a** Non-metric multidimensional scaling (NMDS) based on Bray–Curtis distances among samples of expressed Clusters of Orthologous Groups (COG) (stress = 0.08, PERMANOVA R^2^ = 0.15, F_(1, 8)_ = 1.42, *p-value* = 0.26). Ellipses represent 95% confidence intervals. **b** Boxplots representing the log_2_FoldChange across COG categories with differentially expressed ORFs (adjusted *p-value* < 0.1), which are depicted as red dots

Based on the number of assembled transcripts and genes, and assembly length, the best assembly was version 1 from Trinity (Table S1). After filtering by expression level, we kept fungal ORFs with at least one COG assignment, retrieving 92,293 (41.5%) of the 222,060 predicted ORFs. Among these ORFs, 57,345 were observed in needles of both symptomatic and asymptomatic trees, 16,277 were exclusive of symptomatic needles and 13,173 of asymptomatic needles. Despite these differences, no distinct clustering was observed in COG categories between symptomatic and asymptomatic needles (NMDS stress = 0.8, Fig. 4a). This was supported by a betadispersion analysis, which indicated nonsignificant differences in dispersion between needle conditions (PERMDISP F_(1, 8)_ = 0.7, *p-value* = 0.4). Additionally, the overall composition of COGs did not exhibit differences between conditions (PERMANOVA R^2^ = 0.15, F_(1, 8)_ = 1.42, *p-value* = 0.26). Nevertheless, 30 of the annotated ORFs exhibited significant differences in expression between conditions (adjusted *p value* < 0.10; Fig. 4b); 28 of such ORFs were upregulated in the asymptomatic needles (log_2_FoldChange > 1), and two were downregulated in these same needles (log_2_FoldChange < 1) (Fig. 4b). These ORFs belong to 10 COG categories, the most numerous was “function unknown” (S) (14 ORFs), followed by “carbohydrate metabolism” (G) (6 ORFs); “secondary metabolites biosynthesis, transport and catabolism” (Q) (2 ORFs); and “amino acid transport and metabolism” (E) (2 ORFs). The remaining seven categories only have one ORF (Fig. 4 and Table 1). In turn, eight of the differentially expressed ORFs (ORF 4, ORF 5, ORF 7, ORF 8, ORF 11, ORF 17, ORF 19, and ORF 20) have been previously reported to be upregulated in fungi in response to oxidative stress or as redox systems (Table 1).

**Table 1:** Annotation of significant differentially expressed fungal ORFs between samples from asymptomatic and symptomatic needles of *Abies religiosa* trees

ORF	COG cat.^a^	Pref. name	PFAMs	Metabolic Pathw./ Prot. fam.^b^	Description	Stress	Target species
1	S	-	-	-	-	-	-
2	S	-	Grg1	-	Pfam:DUF2823	Carbon starvation [79]	*Podospora anserina*
3	G	-	MFS_1	SCP	Major facilitator superfamily	Response to toxic compounds [80]	*Colletotrichum higginsianum*
4	GO	-	DUF1996, WSC	-	Yeast cell wall integrity and stress response	Osmotic, oxidative and cell wall perturbation [81]	*Beauveria bassiana*
5	G	GPD	Gp_dh_C, Gp_dh_N	CM, EM, ST	Glyceraldehyde-3-phosphate dehydrogenase	Salinity [82], Oxidative [83, 84]	*Aspergillus sydowii, Saccharomyces cerevisiae*
6	S	-	But2	-	Gpi anchored cell wall	-	*-*
7	S	-	CFEM	-	CFEM domain	Oxidative [85]	*Magnaporthe oryzae*
8	S	-	Ferritin_2	-	Ferritin-like domain	Oxidative [86]	*-*
9	S	-	-	-	-	-	*-*
10	S	-	Peroxidase_2	-	Peroxidase, family 2	Oxidative [87]	*-*
11	E	-	GMC_oxred_C, GMC_oxred_N	AAM	Alcohol oxidase	Oxidative [88]	*Aspergillus nidulans*
12	S	-	GFA	-	Glutathione-dependent Formaldehyde-activating enzyme	-	*-*
13	P	qutD	Sugar_tr	-	-	-	-
14	G	-	GFA, SLT	-	Transglycosylase SLT domain	-	*-*
15	C	ATP1	ATP-synt_ab, ATP-synt_ab_C, ATP-synt_ab_N	EM, EnvA	ATP synthase	-	-
16	Q	-	ADH_N, ADH_N_assoc, ADH_zinc_N	XDM, CM, AAM, EM, LM, MCV	Dehydrogenase	-	-
17	S	-	Peroxidase_2	-	Peroxidase, family 2	Oxidative [87]	*Magnaporthe oryzae*
18	E	-	DHQ_synthase	AAM	3-dehydroquinate synthase	-	-
19	S	-	Ferritin_2	-	Ferritin-like domain	Oxidative [89]	-
20	G	gel4	Glyco_hydro_72, X8	M; GIP; SCP	Beta-glucan production	-	-
21	S	-	Arrestin_C,Arrestin_N	GIP	Arrestin (or S-antigen), C-terminal domain	-	-
22	S	-	DUF4449	-	Protein of unknown function	-	-
23	B	-	DDT, WAC_Acf1_DNA_bd, WHIM1,WSD	-	DDT domain-containing protein	-	-
24	Q	-	FMO-like, Pyr_redox_2	-	Pyridine nucleotide-disulphide oxidoreductase	-	-
25	I	-	Thiolase_C,Thiolase_N	LM, TC, AAM	Belongs to the thiolase family	Heavy metals (Pb) [90] Temperature, Salinity and Ethanol [91]	*Curvularia tsudae, Aspergillus oryzae*
26	G	-	MSF1	SCP	Major Facilitator Superfamily	Response to toxic compounds [80]	*Colletotrichum higginsianum*
27	S	-	DUF1761	-	Protein of unknown function	-	-
28	O	-	Peptidase_S8, Pro-kuma_activ	TC	Pro-kumamolisin, activation domain	Salinity [92]	*Zymoseptoria tritici*
29	S	-	Cutinase	-	Esterase	-	-
30	T	OSH6	Oxysterol_BP	SCP, GIP	Belongs to the OSBP family	-	-

^a^COG categories. S: Function Unknown; G: Carbohydrate transport and metabolism; O: Posttranslational modification, protein turnover, chaperones; E: Amino acid transport and metabolism; P: Inorganic ion transport and metabolism; C: Energy production and conversion; Q: Secondary metabolites biosynthesis, transport, and catabolism; B: Chromatin structure and dynamics; I: Lipid transport and metabolism; T: Signal transduction mechanisms. ^b^Metabolic pathways/ Protein families. SCP: Signaling and cellular process; CM: Carbohydrate metabolism; EM: Energy metabolism; ST: Signal transduction; AAM: Amino acid metabolism; EnvA: Environmental Adaptation; XDM: Xenobiotics biodegradation and metabolism; CM: Carbohydrate metabolism; AAM: Amino acid metabolism; EM: Energy metabolism; LM: Lipid metabolism; MCV: Metabolism of cofactors and vitamins; M: Metabolism; GIP: Genetic information processing; SCP: Signaling and cellular processes; LM: Lipid metabolism; TC: Transport and catabolism. Metabolic pathways correspond to KEGG categories and protein families (underscored) to KEGG BRITE classification. Underexpressed ORFs are highlighted in bold; all remaining ORFs were overexpressed

Only 12 of the 30 ORFs with significantly differential expression had a KO assignment in the KEGG database. The metabolic pathways of these ORFs predicted by the second-level KEGG database included carbohydrate, energy, lipid, amino acid, nucleotide, terpenoids, and polyketides metabolism, as well as biosynthesis of secondary metabolites. As expected, most ORFs are involved in more than one pathway. Additionally, we found that the 12 ORFs with a KO assignment were represented in 91 complete KEGG modules (18.9% of all modules enlisted in KEGG), which were shared by symptomatic and asymptomatic needles. Most of these modules are related to amino acid (26 modules) and carbohydrate metabolisms (19 modules). Two additional modules were exclusive of symptomatic needles, which are related to lipid (β-oxidation) and nucleotide (guanine ribonucleotide degradation) metabolisms.

## Discussion

### Mycobiome from symptomatic and asymptomatic individuals are taxonomically similar

Contrary to our first hypothesis, we did not observe statistically significant differences in the taxonomic composition, observed richness or relative abundance of fungal communities from symptomatic and asymptomatic *A. religiosa* needles when using either ITS2 metabarcoding or RNA-seq metatranscriptomics. Classes Dothideomycetes, Eurotiomycetes and Sordariomycetes were the most abundant in our analyses, regardless of the presence/absence of symptoms or dataset used (Fig. 3). These classes are commonly detected in studies characterizing leaf-inhabiting fungal communities of both angiosperms and gymnosperms, e.g., in *Solanum* [93], *Quercus* [94], *Vitis* [95] and *Abies* species such as *A. koreana* [96]*.* In our study, class Leotiomycetes was marginally more abundant in asymptomatic needles than in those from symptomatic needles when using ITS2 metabarcoding (Fig. 3). This class includes many foliar fungal endophytes that are particularly common in conifers, like *Pinus* and *Abies* [97].

When using the ITS2 metabarcoding dataset, we found only two OTUs that were significantly more abundant in asymptomatic needles, one belonging to genus *Phaeomoniella* and the other one to order Lecanorales. *Phaeomoniella* species include the pathogenic *P. chlamydospora*, which has been associated with Petri and esca diseases in grapevines [98], and the epiphytic and acid-tolerant species *P. zymoides* and *P. pinifoliorum* [99]. Manual BLAST searches in NCBI and UNITE [41] databases using this OTU as query retrieved similar sequences (99.23%–99.61% identity) from unnamed fungal endophytes and *Phaeomoniella* sp. (species hypothesis SH0801475.10FU), all isolated from surface-sterilized pine asymptomatic needles in Arizona and New Mexico, USA. This supports the identification of this OTU, although it likely represents an undescribed species that deserves additional studies. On the other hand, the presence of an OTU belonging to order Lecanorales, a mostly lichenized lineage [100], could indicate a lichen propagule in the needle surface.

The lack of significant differences in guild composition between asymptomatic and symptomatic needles (Fig. S3) is likely the result of a similar fungal community. Although we found greater relative abundance for pathogens, regardless of the needle condition, it should be noted that fungi, particularly endophytes, are known for their ability to switch among lifestyles [5]. Thus, it is essential to acknowledge that it is not possible to distinguish pathogens from endophytes from presence data only. Additional limitations to study mycobiomes from tropical and subtropical plants (reviewed in Narvaez-Trujillo et al. [101]) include that fungi from these regions are underrepresented in the databases used for guild assignment (i.e., UNITE [41]).

Significant differences in mycobiome communities with an enrichment of pathogenic fungi were observed in *Euonymus japonicus* plants experimentally exposed to different ozone concentrations (100–10,000 ppb) [10]. Although O_3_ concentration in Mexico City surroundings can reach peaks as high as 161 ppb [12], sacred fir trees in the field are not as exposed as the plants within a greenhouse experiment, where there is no wind to dissipate air pollutants and, therefore, O_3_ alone may not be the only factor affecting mycobiome composition. Indeed, a landscape-level monitoring of the extent of O_3_ damage within the study area [14], found that O_3_-related symptoms in sacred fir trees are often combined with other environmental factors (e.g. drought and herbivory) [14]. It is likely that the effect of O_3_ on the mycobiome assembly over extended periods of time is relatively less significant than other biotic and abiotic factors, such as host species, plant genotype or spatial distance [102–104]. For instance, grapevine fungal communities are less influenced by pathogen stress than by subtle differences in environmental conditions, such as the edaphic composition, temperature, humidity, and sunlight exposure [105]. Thus, other confounding environmental factors may also explain the absence of differences in observed richness or relative abundance of fungal communities between symptomatic and asymptomatic needles.

It might be argued that our study had a small sample size, and that the species accumulation curve was not saturated (Fig. S4), which implies that increasing the sampling effort could enable the detection of additional fungal species and identify putative differences between symptomatic and asymptomatic needles. Nevertheless, other studies with varying sample efforts and plant systems (e.g., *Eucalyptus*, *Vitis* and *Vainilla*) also failed to detect differences in the fungal communities between plants with different phenotypes (e.g. caused by pathogens; [8, 9, 105]. In addition, we did find a high heterogeneity in the relative abundance of fungal classes across samples (Fig. 3), which may reflect the complexity of factors affecting the assembly of the mycobiome [106]. As more mycobiome studies become available with larger sample sizes and at various landscape levels, it would be possible to disentangle the relative contribution of environmental variables, host genetics and other factors to the host mycobiome composition.

### Foliar mycobiome from asymptomatic needles differentially express genes associated with antioxidant mechanisms

As expected in our second hypothesis, we found 30 putative genes differentially expressed in asymptomatic needles when compared to symptomatic needles (Fig. 4b). Annotated ORFs were usually represented in more than one metabolic pathway, and some were not associated with any pathway (Table 1). We found that eight of these 30 differentially expressed ORFs (ORF 4, ORF 5, ORF 7, ORF 8, ORF 10, ORF 11, ORF 17, and ORF 19) contained domains previously reported in upregulated proteins in response to oxidative stress or redox systems in fungi. For instance, ORF 4 harbors WSC (cell wall stress-responsive component) domains found in the Wsc1I protein, which is known for its role in detecting stress signals in *Beauveria bassiana* [81], including oxidative and osmotic stresses. ORF 5 is similar to a GPD domain-containing protein (glycerol-3-phosphate dehydrogenase), which has been shown to be upregulated in halophilic fungi under high-salinity conditions [82]. Enzymes from this family catalyze the reduction of NADH to NADPH and the reoxidation to NADH, a cycle associated with the defense against reactive oxygen species (ROS) during stressful periods in yeast [83, 84].

Other upregulated ORFs related to oxidative stress in asymptomatic needles (ORF 10 and ORF 17) contained domains from class II peroxidases, which are enzymes that catalyze the oxidation of inorganic and organic substrates using H_2_O_2_ [107]. These peroxidases reduce H_2_O_2_ and can oxidize a wide range of substrates. Their most prominent association is with lignin decomposition in several white-rot fungi [108], although they are also found in some necrotrophic or hemibiotrophic ascomycetes [109]. Indeed, two Class II peroxidases have been previously found to be upregulated in oxidative stress conditions in the pathogenic fungus *Magnaporthe oryzae* [87]. On the other hand, the upregulated ORF 7 contains a CFEM domain that has been found in the Pth11 protein that regulates ROS homeostasis during appressorium formation in *M. oryzae* [85]. Likewise, ORF 11 contains an alcohol oxidase domain that is significantly induced in *Aspergillus nidulans* under long-term exposure to oxidative stress [88].

Further upregulated ORFs (ORF 8 and ORF 19) contained ferritin-like domains. Ferritins are recognized in plants and animals for their role in regulating iron levels within cells, and thus preventing ROS formation [86]. While most fungi lack ferritin enzymes [110], some contain ferritin-like sequences in their genomes, and it is likely that ferritin-like proteins in fungi play a similar role as those in plants and animals [111]. Also related with iron metabolism, another upregulated ORF (ORF 24), containing a Pyr_redox_2 domain, is related to siderophore biosynthesis in *Paracoccidioides brasiliensis* [112]. However, the links between O_3_, iron balance, and siderophores in fungi remain largely unexplored.

We also detected several upregulated ORFs in asymptomatic needles containing domains associated with other stress responses. For example, ORF 2 (Table 1) has a Grg1 domain whose abundance increases during periods of carbon starvation in fungi [79]. Linked to detoxification mechanisms, we found an MFS domain in ORF 3 and ORF 26 [80] and an FMO-like (Flavin-containing monooxygenase) domain in ORF 24 [113]. Two ORFs (ORF 25 and ORF 28) further contain domains related to salinity stress, a pro-kumamolisin activation domain (ORF 28) [92] and two thiolase domains (ORF 25) [91]. Such thiolase domains are associated with resistance to high temperatures and ethanol stress in *Aspergillus oryzae* [91], and heavy metal stress, particularly lead, in *Curvularia tsudae* [90].

Although some upregulated ORFs (ORFs 20 and 22) could not be directly linked to a specific stressor, they contained domains that could be relevant in other cellular processes. For example, ORF 20 contains two domains present in protein gel4, which is relevant for maintaining cell wall integrity in some fungi [114]. Proteins with this domain can also modify cell walls facilitating plant host infection by fungal pathogens [114]. Likewise, ORF 22 contains domain DUF4449 that is part of the TmHam13 protein of *Talaromyces marneffei*, which is related to cell differentiation (dimorphism) and signaling transduction during pathogenesis in humans [115].

From the two downregulated ORFs found in asymptomatic needles, one (ORF 21) contained two arrestin domains. Arrestins are a family of proteins that play a role in nutrient transport and signaling receptor functions [116]. In fungi, arrestins have been linked to the regulation of several cellular responses. For example, in *Cryptococcus neoformans*, arrestins are involved in controlling cytokinesis, lipid production for cellular membrane formation, and virulence potential [117]. In *Aspergillus nidulans* and *Arthrobotrys oligospora*, arrestins are further involved in pH signaling [118, 119]. In *A. oligospora*, arrestins are also involved in lifestyle switching (from saprotroph to nematophagous) and conidial phenotype changes, nuclear distribution within cells and stress resistance [117, 119]. The other downregulated ORF (ORF 30) in asymptomatic needles contains an OSH6 domain (Oxysterol-binding protein), which has been associated with oxidative burst, cell death, and plant defense triggered after *Magnaporthe oryzae* infection [120].

Although the proportion of fungal RNA reads was minimal (2–5%) compared to that of the host, we were able to detect several metabolic modules associated with primary pathways, such as carbohydrate, energy, lipid, nucleotide, and amino acid metabolisms. Differences in metabolic pathways specifically associated with needle condition were not observed (Fig. 4a), but two complete metabolic modules were exclusively found in the symptomatic needles: β-oxidation (lipid metabolism) and guanine ribonucleotide degradation (nucleotide metabolism). The β-oxidation module allows the use of lipids as a carbon source [121] and is necessary for cell wall synthesis and turgor generation in the infection structures of some fungal pathogens [122]. In turn, blocking the guanine ribonucleotide degradation module affects the pathogenicity of *M*. *oryzae* and *Fusarium graminearum* [123].

**Fig. 5:**
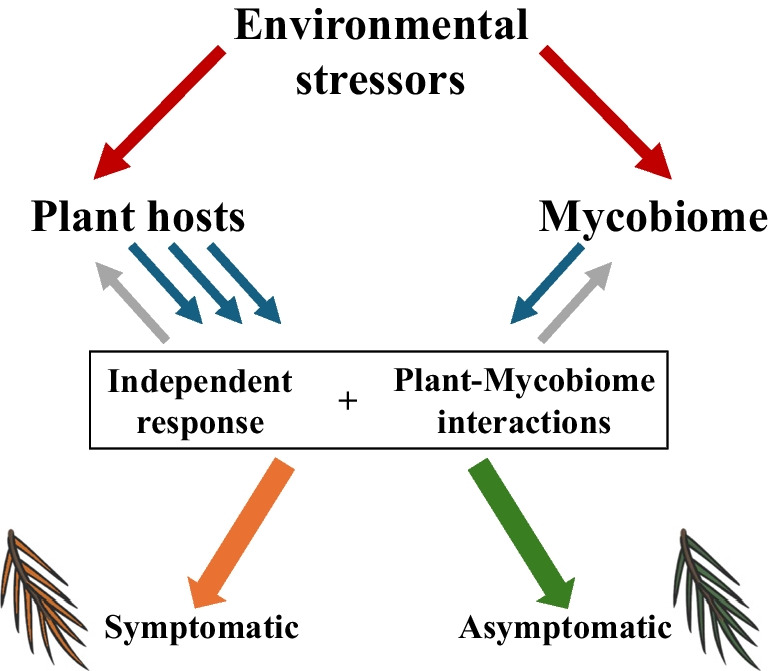
Hypothetical framework for representing the interaction between host plants, their mycobiome and the environment, resulting in two distinct needle phenotypes. Blue arrows represent differential gene expression from different plant individuals (exemplified by three arrows) and the mycobiome. Gray arrows depict potential feedback to both symbionts, resulting from plant-mycobiome interactions

Despite their low number (30), all differentially expressed ORFs seem to be part of metabolic pathways that might be important to counter O_3_ exposure, including oxidative stress, iron homeostasis, maintenance of cell integrity, detoxification, and plant pathogenesis. This is similar to the transcriptomic responses observed for asymptomatic host trees, whose upregulated transcripts were involved in stress response, stomatal opening modulation and the production of secondary metabolites associated with oxidative stress [24]. Differential genetic response in two similar fungal communities could be the result of complex interactions with the plant hosts [124–127]. Environmental stressors, such as O_3_, may indirectly influence mycobiome response by affecting plant defense mechanisms [128]. This is supported by the host trees of this study, which differentially expressed chitinases and LRR protein kinases during the high peaks of O_3_ concentration [24, 129]; all these proteins play important roles in recognizing and responding to pathogens in plants [130]. On the other hand, some studies have demonstrated that fungal endophytes can induce differential gene expression when their host plants are under stress, which may in turn affect the functionality of the fungi themselves [127]. Future studies should also increase sampling and RNA sequencing efforts for delineating more complete metabolic pathways and help us better understand how the interaction between the plant and mycobiome metabolisms increase the tolerance to pollution-related stress. For the particular case of *Abies religiosa*, they could help us test whether changes in the mycobiome metabolism can benefit the host plant by maintaining the functionality of photosynthetic tissues within asymptomatic needles. This may imply an evolutionary advantage for both the plant and its fungal community and indicate that the mycobiome represents a significant selective pressure for plant genotypes. In Fig. 5 we summarize a hypothetical framework for guiding future studies on the relationship between environmental stressors and plant-foliar mycobiome interactions. When faced with an environmental stressor (i.e. O_3_) both plants and mycobiome differentially express genes that may have direct and independent effects on the plant phenotype (i.e. presence/absence of symptoms), but it is likely that two-way feedback plant-mycobiome interactions also influence host phenotypes. Although the precise mechanisms are yet to be discovered, these may include epigenetic factors either produced by the plant influencing the mycobiome response [124, 125] or elicited by the fungal community with effects on the plant hosts [127, 131], as well as intraspecific genotype variation of both plant and fungi [132], among others [8].

### RNA-Seq as a tool for plant mycobiome studies

Our study illustrates that it is possible to use the host RNA-Seq data available from global sequence repositories to characterize the mycobiome and gene expression. However, since there is no standardized workflow for analyzing fungal sequences, a careful interpretation of the results and fine-tuned methodologies are needed to address specific questions. The most important bottleneck for using RNA-seq data from plant studies to characterize the taxonomic composition and function of fungal communities is the minimal proportion of fungal RNA recovered compared to the host, which limits the amount of recovered information, the power of statistical analyses and, therefore, the conclusions drawn from these analyses. For instance, our fungal RNA-seq data only represent between 2 and 5% of all transcripts, but even such low percentages allowed us to characterize the *Abies religiosa* mycobiome and their functions.

Similar to the DNA metabarcoding dataset, we did not find significant differences in the observed richness or relative abundance (Fig. 3) of fungal classes or guilds between symptomatic and asymptomatic needles based on RNA-Seq data. Although the communities recovered using each dataset were different (Fig. 2b), the most representative classes coincided between datasets (Fig. 3). The differences observed could be attributed to the database used to assign taxonomic ranks in each pipeline. For instance, when we examined fungal classes exclusive to the RNA-Seq metatranscriptomics dataset, we found that they comprised predominantly model taxa, such as *Saccharomyces* or *Aspergillus*, indicating a potential bias in classification. A potential way to circumvent this problem is to use transcripts of the ITS region to identify fungi from RNA-Seq data using ribosomal depletion or by direct RNA sequencing of ITS transcripts, as performed in previous studies [133, 134]. Library construction in our study excluded non-polyadenylated transcripts, like structural ribosomal RNAs (rRNA), including the ITS region; this hampered us from retrieving ITS transcripts. The ITS region is the universal fungal barcode [135] and it is widely used for the taxonomic identification of fungi using metabarcoding [38]. Therefore, these transcripts would have been very useful for classifying sequences more accurately with the already available reference databases (i.e., UNITE [41]). In addition, ITS read numbers would have allowed us to quantify fungal abundances, a task that is currently unreliable using PCR-based methods [136], although caution needs to be taken given that there are differences in ITS copy numbers among fungal species [39].

Our functional profiling allowed us to uncover some modules associated with the host phenotypes. These include modules associated with pathogenic behavior, which were observed in symptomatic needles as well as the expression of genes putatively related to the attenuation of oxidative and other types of stress in asymptomatic needles. Increasing RNA sequencing effort will eventually allow the detection of more metabolic modules and more specific genes related to our question. New laboratory methods to enrich the microbial representation of transcripts in metatranscriptomic studies are thus needed, but while these become available, improvements could be made in the bioinformatic methods. For instance, filtering the transcripts using microbial databases, instead of using the hosts as reference, with databases that integrate information from environmental taxa, like JGI Mycocosm [137]. This kind of method has been previously used in a metatranscriptomic study of plant-associated fungi [138], but comparisons between host-filtered and microbial-filtered transcripts are still needed to evaluate which method is better or if they are complementary.

## Conclusions

Tropospheric ozone affects plants at several levels, including the gene expression of their associated mycobiome. We characterized the mycobiome in symptomatic and asymptomatic needles of *A. religiosa* trees from a peripheral forest next to Mexico City that has been heavily exposed to O_3_ for over 40 years [12]. Our results revealed an intriguing pattern: while symptomatic and asymptomatic needles harbor similar fungal communities, fungal genes associated with oxidative stress response are upregulated in fungi from asymptomatic needles, which suggests that the mycobiome responds to the oxidative stress triggered by O_3_. This response, together with the one expressed by their host plants, produce different phenotypes in the same plant population that could be advantageous. However, further studies are still needed to demonstrate that fungal communities may affect and even enhance the host performance when O_3_ concentrations increase. These studies should include controlled experiments, where the effects of other co-occurring pollutants can be accounted for. Future research exploring co-transcription networks between host and fungal communities could further help to infer correlations between host plant and mycobiome responses to O_3_ stress. Finally, we showed that it is possible to use plant RNA-seq metatranscriptomics to gain insights into plant-inhabiting fungi and their response to stress; such approaches have limitations that must also be addressed in future studies.

### Supplementary Information

Below is the link to the electronic supplementary material.

Supplementary file1 (PDF 634 KB)

## Data Availability

No datasets were generated or analysed during the current study.
