# Supplementary material for: Foliar mycobiome remains unaltered under urban air-pollution but differentially express stress-related genes
Source: Microb Ecol. 2024 May 17;87:72. doi: 10.1007/s00248-024-02387-y (PMC11098924; doi:10.1007/s00248-024-02387-y)
Supplement: Supplementary file 1 [file 248_2024_2387_MOESM1_ESM.pdf]

Supplemental information Foliar mycobiome remains unaltered under urban air-pollution but differentially express stress-related genes

Microbial Ecology

Valeria Stephany Flores-Almaraz[a,b]

Camille Truong[c]

Diana Hernández-Oaxaca[d]

Verónica Reyes-Galindo[e]

Alicia Mastretta-Yanes[f,g]

Juan Pablo Jaramillo-Correa[e]

Rodolfo Salas-Lizana[h*]

[a] Posgrado en Ciencias Biológicas, Unidad de Posgrado, Edificio A, 1° Piso, Circuito de Posgrados, Ciudad Universitaria, Coyoacán, C.P. 04510, Distrito Federal, México

[b] Instituto de Biología, Universidad Nacional Autónoma de México, Av. Ciudad Universitaria 3000, 04510, Coyoacán, Ciudad de México, Mexico

[c] Royal Botanic Gardens Victoria, Birdwood Ave, Melbourne, VIC 3004, Australia

[d] Centro de Ciencias Genómicas, Universidad Nacional Autónoma de México, Av. Universidad S/N, 62210. Cuernavaca, Morelos, México

[e] Depto. de Ecología Evolutiva, Instituto de Ecología, Universidad Nacional Autónoma de México, Av. Ciudad Universitaria 3000, 04510, Coyoacán, Ciudad de México, Mexico

[f] Consejo Nacional de Humanidades Ciencias y Tecnología (CONAHCYT), Avenida Insurgentes Sur 1582, Crédito Constructor, Benito Juárez, Ciudad de México. C.P. 03940. México

[g] Comisión Nacional para el Conocimiento y Uso de la Biodiversidad (CONABIO). Avenida Liga Periférico-Insurgentes Sur 4903. Colonia Parques del Pedregal, Tlalpan, Ciudad de México. CP 14010. México

[h] Laboratorios de Micología. Depto. de Biología Comparada, Facultad de Ciencias. Universidad Nacional Autónoma de México, Circuito Exterior s/n, Ciudad Universitaria, Coyoacán, 04510. Ciudad de México, México

*Corresponding authors:

Rodolfo Salas-Lizana: rsalas@ciencias.unam.mx

Alicia Mastretta-Yanes: amastretta@conabio.gob.mx

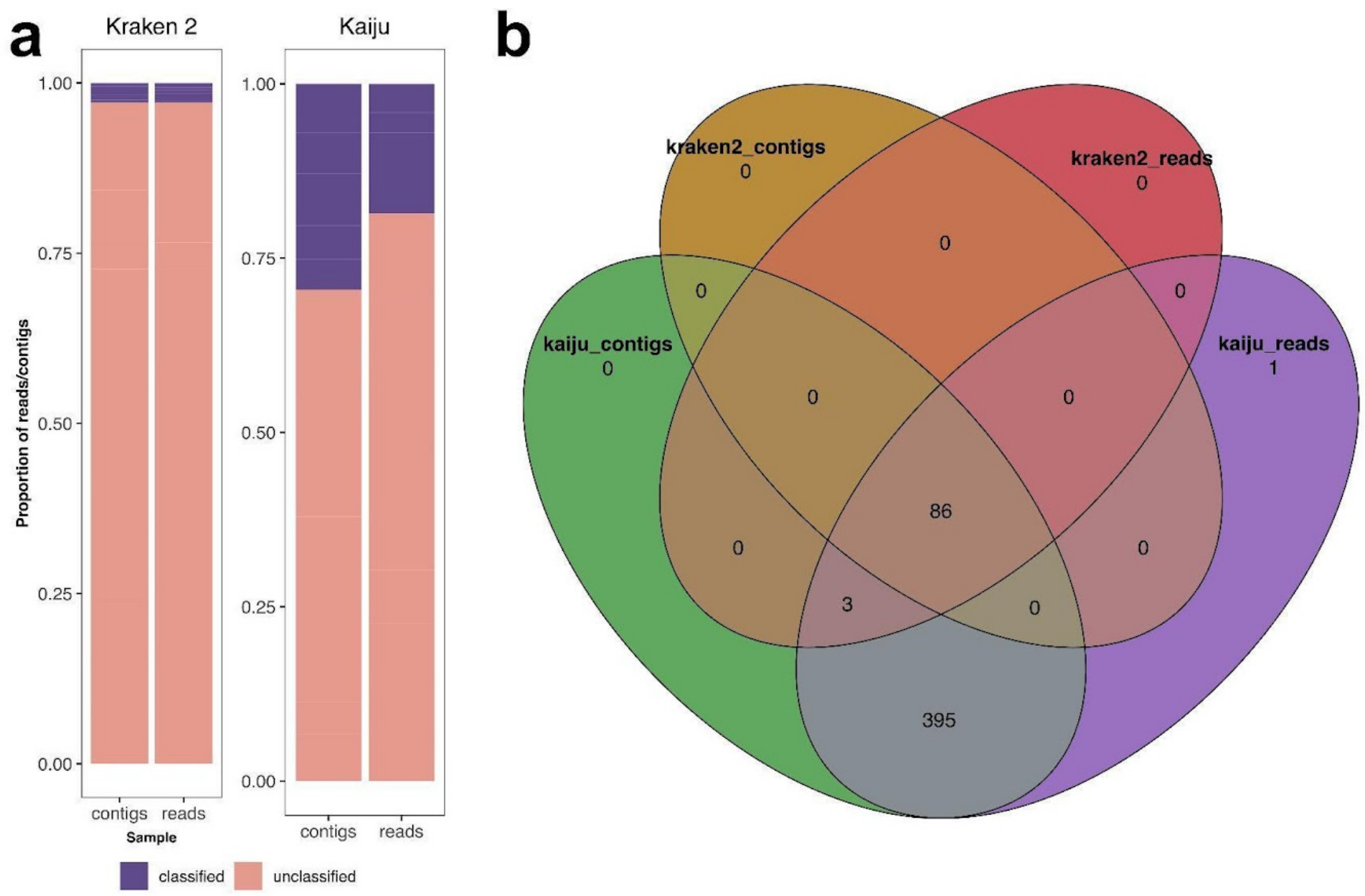

Fig. S1 Taxonomic classification of RNA-Seq data **a** Proportion of classified transcripts per classifier (Kraken2-Bracken or Kaiju) and type of input data (reads or contigs). **b** Number of shared OTUs per classifier (Kraken2-Bracken or Kaiju) and type of input data (reads or contigs).

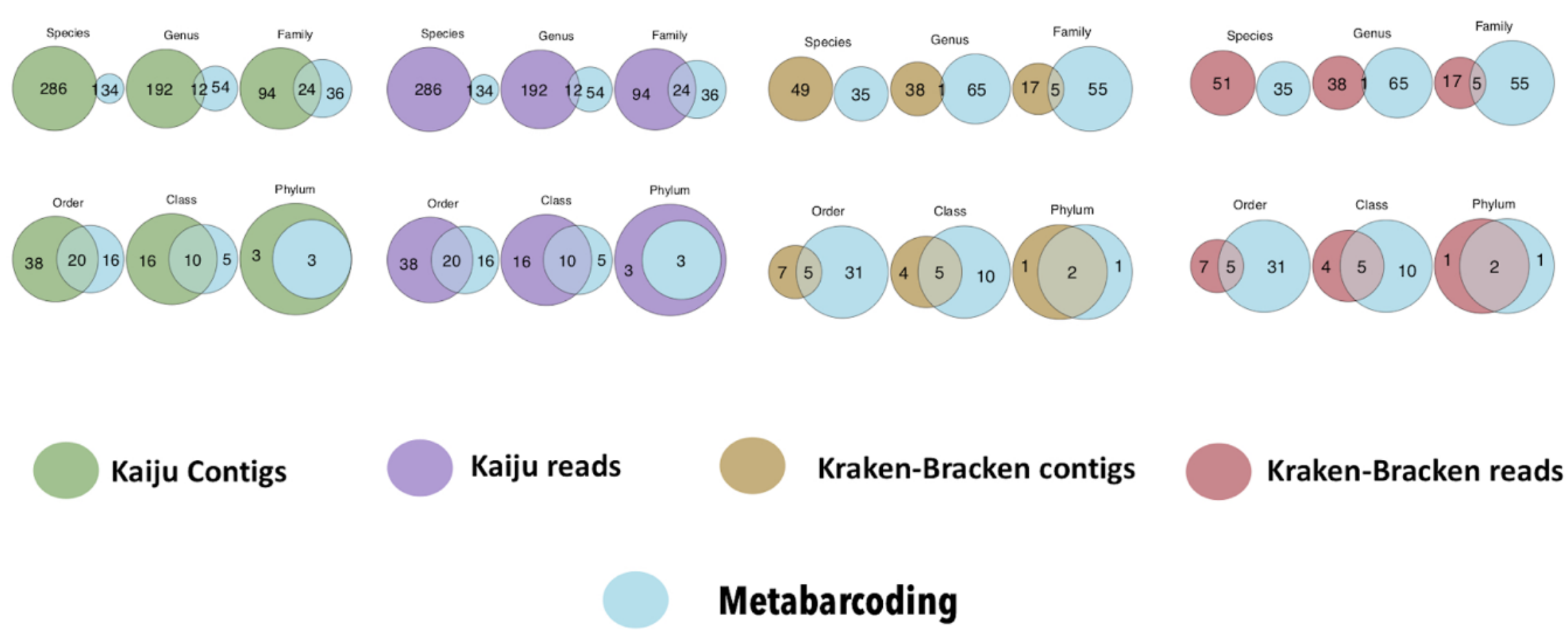

Fig. S2 Venn diagrams of classified OTUs at various taxonomic levels comparing RNA-Seq metatranscriptomic (Kaiju and Kraken2-Bracken, using reads or contigs) and ITS2 metabarcoding data.

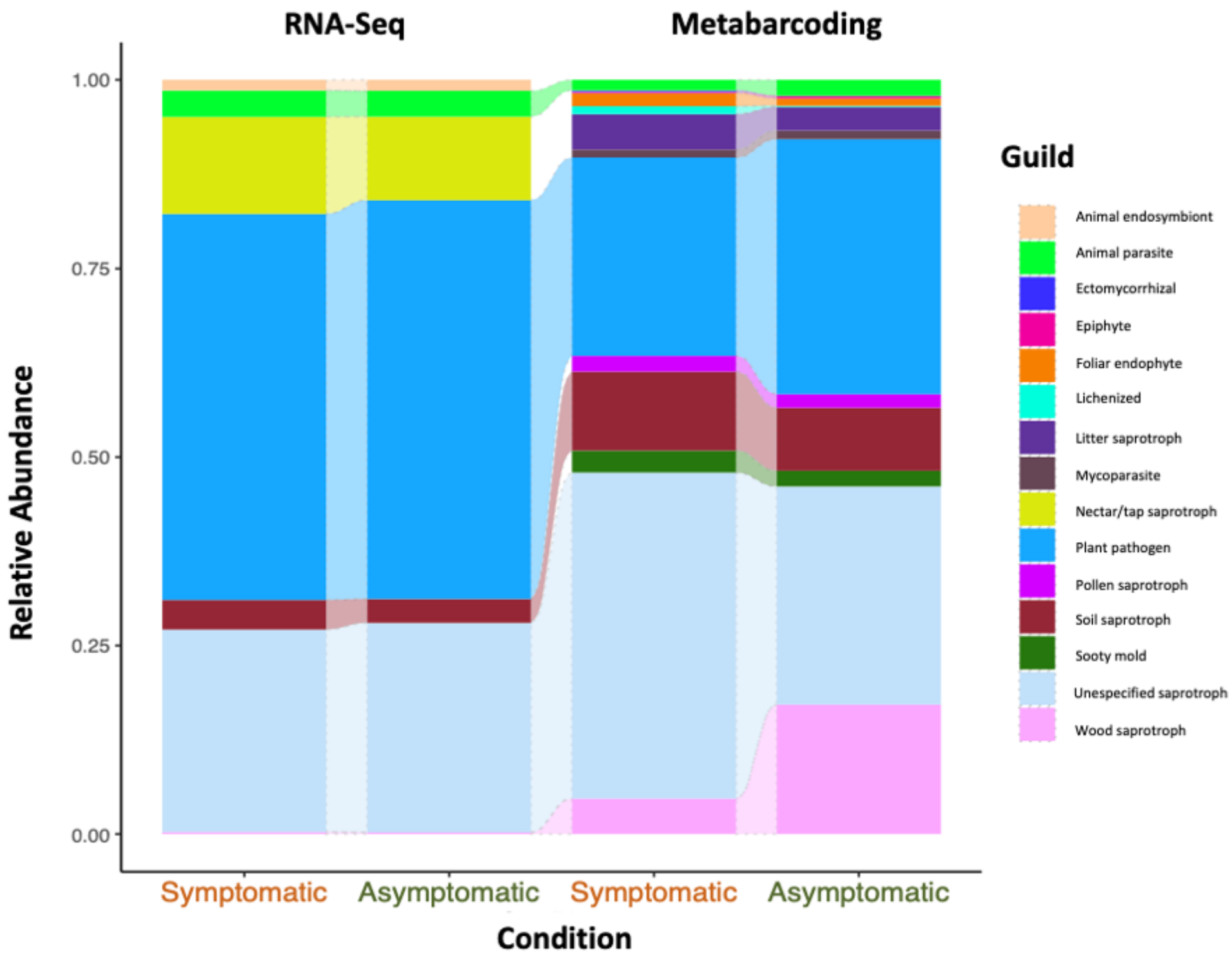

Fig S3. Relative abundance of classified fungal guilds of symptomatic or asymptomatic needles using RNA-Seq metatranscriptomics or ITS2 metabarcoding

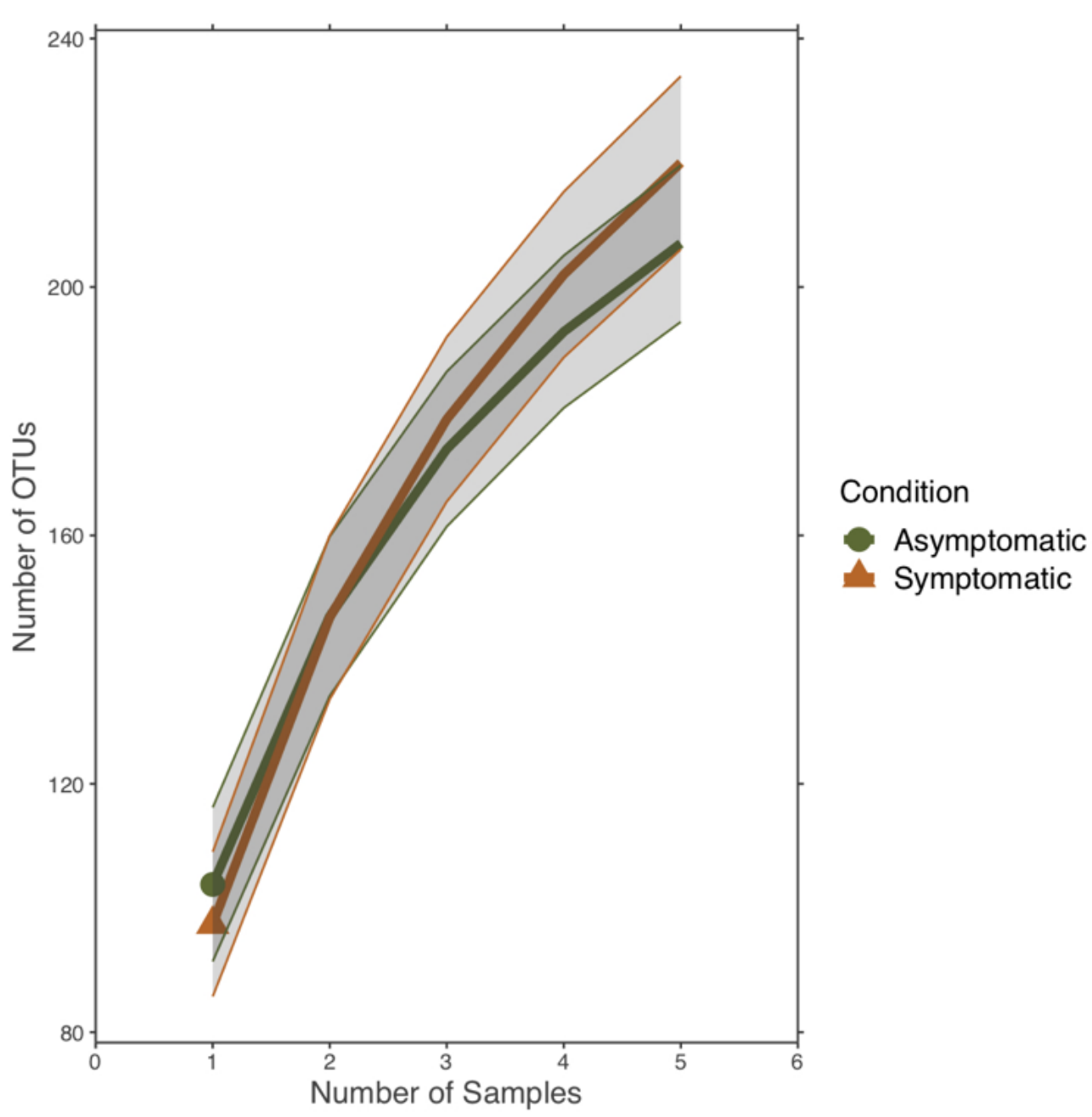

Fig. S4 Species accumulation curve for the ITS metabarcoding dataset by needle condition (symptomatic and asymptomatic).
